# Empowerment or burden? The impact of empowering leadership on Generation Z knowledge workers retention willingness

**DOI:** 10.3389/fpsyg.2025.1706788

**Published:** 2025-11-24

**Authors:** Genlin Zhang, Yichuan Wang

**Affiliations:** School of Management, Xi’an University of Science and Technology, Xi’an, China

**Keywords:** empowering leadership, Generation Z knowledge workers, retention willingness, perceived organizational support, emotional exhaustion

## Abstract

**Instruction:**

In the context of sustainable human resource management, this study investigates the dual-pathway mechanism through which empowering leadership influences the retention willingness of Generation Z knowledge workers in small and medium-sized enterprises (SMEs), based on the Conservation of Resources (COR) theory. We propose that perceived organizational support and emotional exhaustion serve as parallel mediators, while proving and avoidance goal orientations act as critical moderators.

**Methods:**

A multi-industry survey was conducted, collecting valid data from 387 Generation Z knowledge workers across various sectors in China. Hypotheses were tested using structural equation modeling (SEM) to analyze the direct, mediating, and moderating effects.

**Results:**

The findings revealed that empowering leadership exerts an ambivalent effect on retention willingness. It simultaneously increases retention willingness by enhancing perceived organizational support and decreases it by exacerbating emotional exhaustion, with both mediating paths being statistically significant. Furthermore, employees’ goal orientation moderates this mechanism: proving goal orientation strengthens the positive effect of empowering leadership on perceived organizational support, whereas avoidance goal orientation intensifies its positive effect on emotional exhaustion.

**Discussion:**

This study unveils the “double-edged sword” effect of empowering leadership, providing a more comprehensive and dialectical perspective on its impact on employee retention. The findings offer valuable insights for SMEs to implement sustainable human resource practices by tailoring empowering leadership based on employee characteristics to enhance retention and foster organizational sustainability.

## 1 Introduction

The transition toward a sustainable knowledge-driven economy has shifted organizational competition from traditional resource acquisition to talent retention, making effective human capital management a critical challenge (Zhang and Wang, 2017). As a vital economic entity, small and medium-sized private enterprises (SMEs) face particular difficulties in sustainably retaining Generation Z knowledge workers (born 1995–2009), a cohort distinguished by unique values, work attitudes, and career expectations that significantly influence organizational innovation and competitiveness. However, their “high turnover rate,” “frequent job-hopping,” and “low engagement” also threaten the sustainable growth of SMEs (Jamil et al., 2025).

Existing research points out that job satisfaction negatively predicts turnover intention among knowledge workers (Hofaidhllaoui and Chhinzer, 2014). To enhance retention willingness, scholars suggest designing jobs aligned with Generation Z values to increase their perceived sense of meaning (Popaitoon, 2022). In addition, some scholars propose employing transformational and empowering leadership styles, emphasizing delegation and attentiveness to employees’ psychological states, to meet the higher-level psychological needs of the new generation workforce and improve job satisfaction (Zhang and Wang, 2017). Since empowering leadership has a positive impact on employee retention decisions by promoting employees’ emotional commitment to the organization, organizations can reduce employee turnover and improve employee retention rates by cultivating empowering leadership (Cheng, 2019; Kim and Beehr, 2020).

However, most of the current research on employee retention willingness or turnover tendency from the perspective of empowering leadership is limited to a single perspective and fails to comprehensively consider the complex relationship between empowering leadership and employee retention willingness. Moreover, mainstream research generally emphasizes the positive role of empowering leadership, implying that “empowerment is always good for employees” (Hao et al., 2014; Chen et al., 2011). Although some scholars have raised doubts and conducted research on the “dark side” of empowering leadership, suggesting that positive leadership behaviors can also yield negative consequences, research constructing dual-process mechanism models to comprehensively explore its positive and negative impacts on retention intention remains insufficient (Cheong et al., 2016). There is a particular lack of in-depth investigation focusing on Generation Z knowledge workers within SMEs.

Therefore, based on the conservation of resources (COR) theory, this study explores the dual-process mechanism of empowering leadership on the retention willingness of Generation Z knowledge workers, with goal orientation as the boundary condition of this process mechanism. Accordingly, the following research questions are proposed: (1) Does empowering leadership affect the retention willingness of Generation Z knowledge workers? (2) What is the process of empowering leadership on the retention willingness of Generation Z knowledge workers? (3) What are the boundary conditions of the influence of empowering leadership on the retention willingness of Generation Z knowledge workers? This study will test hypotheses by constructing a structural equation model, aiming to provide a more comprehensive and dialectical perspective on empowering leadership and Generation Z employee retention, revealing the mechanisms underlying its positive and negative effects.

## 2 Theoretical foundation and research hypotheses

This study adopted COR as its foundational framework. This theory posits that individuals strive to retain, protect, and accumulate resources they value (e.g., time, energy, abilities, social support, etc.) and that individuals are more sensitive to resource losses (Hobfoll, 1989; Hobfoll et al., 2018). The theory’s key dynamic mechanisms are embodied in the resource gain spiral: individuals with abundant resources are more capable of acquiring new resources and willing to invest resources, meaning resource accumulation leads to further resources. Conversely, the resource loss spiral: individuals lacking resources tend to activate a defensive mode to protect against resource loss, meaning resource depletion triggers stress responses, leading to negative emotions and behaviors (Zhang et al., 2023; Li et al., 2022). These two spiral effects explain how resource status influences individual behavior and outcomes, and research indicates that resource loss exacerbates burnout, while resource acquisition alleviates burnout (Hobfoll et al., 2018). Chinese scholars Cao and Qu (2014) through a systematic review of COR’s origins, development, and core content, derived three interrelated propositions: the primacy of resource conservation, the secondary nature of resource acquisition, and the creation of resource surplus.

Additionally, COR theory has been applied in multiple domains such as stress, job burnout, performance, and organizational commitment. Researchers building on COR developed the Job Demands-Resources (JD-R) model from a work characteristics perspective. Applying the JD-R model revealed that resource factors significantly predict job burnout that resource factors significantly predict work (Fu et al., 2008).

By introducing Goal Orientation (GO), this study explores how empowering leadership behaviors influence the cognitive mechanisms of Generation Z knowledge workers and how cognitive appraisals shape their understanding and response to role stress. As a typical individual trait, GO varies among people, reflecting different goal preferences in achievement situations (Payne et al., 2007). GO includes learning GO and performance GO, with the latter further divided into prove performance GO and avoid performance GO. Learning GO reflects an individual’s desire to increase knowledge and skills. Prove performance GO describes an individual’s wish to demonstrate competence and outperform others, while avoid performance GO represents an individual’s wish to avoid appearing incompetent in achievement pursuits (Downes et al., 2021). This study focuses on the two performance-related orientations because leaders engage in delegating behavior to help employees better use delegated authority for self-management, task goal setting, and decision-making participation, thereby improving work performance.

### 2.1 Empowerment mechanisms of empowering leadership

Perceived organizational support (POS, also known as employer commitment) is an employee’s subjective perception of whether the organization values their contributions, cares about their welfare, and supports their work and development (Chen, 2006). Research demonstrates that POS significantly positively influences employee retention intention (Cropanzano et al., 1997). As organizational structures become flatter and decision-making power becomes more decentralized, teamwork and communication become increasingly crucial, making empowering leadership, which emphasizes teamwork and communication, key to adaptive management.

Ahearne et al. (2005) conceptualized empowering leadership across four dimensions: enhancing the meaningfulness of work, fostering participation in decision-making, expressing confidence in high performance, and providing autonomy. According to COR theory’s gain spiral effect, when employees need empowering resources from leaders, they actively invest these resources into work tasks to maximize their utility, thereby enhancing employees’ self-efficacy, thriving at work, and perceived organizational support. With more resources, employees gain opportunities to create further advantageous resources, increasing their willingness to remain with the organization (Guo and Wang, 2014; Xiang et al., 2020).

Generation Z knowledge workers, typically highly educated with strong independent thinking and self-directed learning abilities, experience enhanced POS from such empowering behaviors. By clearly articulating work meaning and objectives, employees better understand how their work connects to the organization’s overall goals and values. When employees comprehend their work’s importance to the organization, they are more likely to feel supported and recognized, thereby enhancing POS. Encouraging employee participation in decision-making demonstrates leaders’ respect for their opinions and professional capabilities. This participation fosters a sense of belonging and responsibility, significantly increasing loyalty and POS toward the organization.

In addition, leadership trust in employees’ high performance is a key factor in motivating employees, and the autonomy given to employees allows them to complete tasks at their own pace, thereby enhancing their sense of organizational identity and support. Since Generation Z knowledge workers value opportunities for personal growth and development, they are more likely to strengthen their commitment to the organization when they feel that the organization invests resources and attention in their work and career development (Eisenberger and Armeli, 1997). At the same time, as digital natives, they care about work-life balance and the organization’s concern for their welfare. Therefore, by providing flexible work arrangements, health benefits, and attention to employees’ individual needs and issues, organizations can improve employee satisfaction and loyalty, thereby increasing retention willingness.

In summary, for Generation Z knowledge workers, high levels of POS satisfy their emotional needs and desire for autonomous work, strengthening their affective commitment and sense of belonging to the organization. They become more loyal, increase their work engagement, and are more inclined to remain with the organization. Based on this, the following hypothesis is proposed:

*Hypothesis 1*: Empowering leadership positively affects perceived organizational support, and perceived organizational support positively affects the retention willingness of Generation Z knowledge workers. Furthermore, perceived organizational support mediates the relationship between empowering leadership and the retention willingness of Generation Z knowledge workers.

### 2.2 The burden mechanism of empowering leadership

Although empowering leadership is considered to enhance employee autonomy, engagement, and job satisfaction, it can also yield negative consequences under certain circumstances, particularly in environments where employees prefer directive leadership. Emotional exhaustion is the feeling of emotional resource depletion due to the excessive consumption of emotional resources at work (Lu et al., 2013; Maslach and Jackson, 1981). Emotional exhaustion has a negative impact on employees’ organizational commitment and job satisfaction. It can cause employees to doubt their own work abilities and achievements, feel ineffective and powerless at work, and develop negative emotions such as boredom, anger, or indifference, which in turn affect their work attitude and job performance (Wright and Cropanzano, 1998). Current research on emotional exhaustion predominantly involves helping professions like university teachers, healthcare workers, and police officers (Zhang et al., 2021), with limited studies focusing on emotional exhaustion among Generation Z knowledge workers.

According to COR theory, when employees do not need authorized resources to complete a task, authorization may be seen as a potential risk and become a “burden.” This empowerment implies increased workload and task difficulty. Employees may feel pressured by the added decision-making responsibility, consuming significant time and emotional resources to fulfill tasks. Consequently, when they receive empowering resources, their emotional resources are heavily depleted, leading to a state of emotional exhaustion. To prevent further resource loss, they may adopt negative work behaviors such as passive resistance or tardiness, exemplifying COR’s loss spiral effect (Sut et al., 2014).

Research on emotional exhaustion and employee retention intention examines the relationship between emotional exhaustion and turnover intention across various professions. Ran et al. (2020) found that work exhaustion and turnover tendency were positively correlated, and that work exhaustion may indirectly increase turnover intention by reducing job satisfaction. Zhang et al. (2021), investigating nurses’ job burnout and turnover intention in tertiary hospitals, reached similar conclusions: nurses’ job burnout significantly influenced turnover intention, with higher burnout correlating with stronger turnover intention. They identified emotional exhaustion as the core dimension of job burnout and a key indicator of emotional resource depletion.

In summary, due to the complexity and challenges of working conditions and tasks, for Generation Z knowledge workers, long-term exposure to high-intensity work may indeed lead to emotional exhaustion, which in turn may cause them to want to leave the organization. Emotionally exhausted employees are more likely to reduce work engagement and satisfaction, increase job-seeking behavior, and exhibit a series of negative behaviors, which in turn may reduce their retention willingness and increase the likelihood of employee turnover. Based on this, the following hypotheses are proposed in this study:

*Hypothesis 2*: Empowering leadership positively affects emotional exhaustion, emotional exhaustion negatively affects the retention willingness of Generation Z knowledge workers. Furthermore, emotional exhaustion mediates the relationship between empowering leadership and the retention willingness of Generation Z knowledge workers.

### 2.3 The moderating effect of proving goal orientation

According to the COR theory, leadership empowerment is regarded as a resource necessary for employees to complete tasks; leaders provide this resource through interaction with employees. Employees needing the resource, upon receiving empowerment, invest it better to produce resources, forming a gain spiral. Based on COR’s fundamental tenet, when facing the stimulus of leader empowerment, whether an employee needs the resource supply depends on their cognitive appraisal (Song et al., 2023). Different employees will have different cognitive assessments when faced with delegation. For Generation Z knowledge workers with a prove performance GO, characterized by traits like actively accepting challenges, self-motivation, and self-improvement, they tend to focus on leader-provided empowering resources when completing organizational tasks. They have a high demand for leader empowerment, viewing it as crucial for their own success and goal achievement. This enables them to better manifest self-management leadership, thereby enhancing work performance. Consequently, they actively accept leader empowerment, perceiving it as an opportunity for self-development and success (Koopmann et al., 2019), and believe supervisors support their work and value their capabilities and contributions, thereby enhancing POS. Simultaneously, empowering leadership behaviors help prove-oriented employees achieve goals, making them less likely to feel the extra burden and pressure associated with leader empowerment.

In summary, based on COR, for Generation Z knowledge workers with a prove performance GO—who are more independent, self-aware, and seek empowerment to demonstrate and showcase their abilities—there is a higher demand for empowering resources from leaders. By acquiring empowering resources, they invest resources more effectively, yielding positive work behaviors and self-efficacy, leading to more positive organizational perceptions that enhance POS and satisfaction. Based on this, the following hypothesis is proposed:

*Hypothesis 3*: Proving goal orientation has a positive moderating effect on empowering leadership and perceived organizational support. Specifically, the higher the prove performance goal orientation, the stronger the relationship between empowering leadership and perceived organizational support among Generation Z knowledge workers, and vice versa.

### 2.4 The moderating effect of avoidance goal orientation

Based on COR theory, when employees do not need empowerment resources, they may fall into a spiral of resource loss, leading to psychological resource depletion and emotional exhaustion. The occurs because some employees rely more on leaders providing specific requirements and instructions to execute tasks; they inherently prefer not to gain autonomy or manage themselves by setting their own goals. In such cases, leader empowering behaviors are not perceived as “enabling” but rather as a “burden” (Sut et al., 2014). For Generation Z knowledge workers with an avoid performance GO, characterized by a tendency to avoid risks, avoid failure, and focus on completing tasks safely, they prefer to depend on direct instructions and guidance from superiors rather than making independent decisions and setting goals themselves. Therefore, when faced with the delegated resources of leaders, they view them as a form of pressure, which reduces their POS and organizational commitment. Additionally, they focus more on the potential negative outcomes of tasks, spending significant time and effort preparing to avoid any possible errors or failures (Dong et al., 2023).

In summary, based on COR and role theory, when faced with empowering leadership resources, Generation Z knowledge workers with avoidance GO will view them as a pressure or challenge and will be more inclined to avoid risk and failure. Moreover, conflict between their own role perception and leaders’ role expectations can generate work pressure and emotional exhaustion, leading to further loss of emotional resources. Based on this, the following hypothesis is proposed:

*Hypothesis 4*: Avoidance goal orientation has a positive moderating effect on empowering leadership and emotional exhaustion. The higher the avoidance GO, the stronger the relationship between empowering leadership and emotional exhaustion among Generation Z knowledge workers, and vice versa.

**Figure 1:**
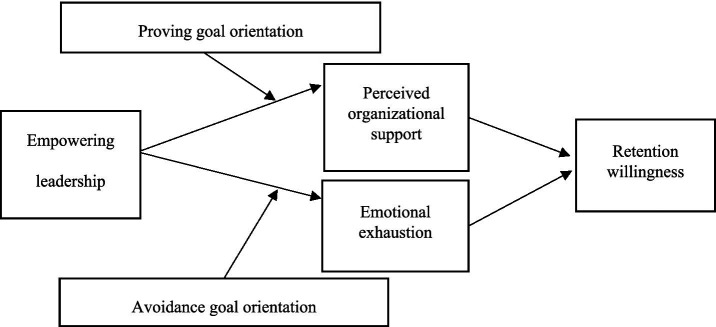
Theoretical model diagram.

In summary, the theoretical model of this study is shown in Figure 1.

## 3 Research design

### 3.1 Research participants and survey process

This study defines Generation Z knowledge workers as individuals born in 1995 or later, currently employed, holding an associate degree or higher, who utilize knowledge and information in their work, possess professional expertise in their field, exhibit strong learning abilities and high self-awareness, and value personal career development prospects. Data were collected using scaled questionnaires. Participants meeting the criteria were surveyed within small and medium-sized private enterprises located in high-tech industrial development zones in Shaanxi, Sichuan, and Beijing, China. The sample covered industries including information technology and internet, services, manufacturing, and finance, ensuring representativeness. To avoid the impact of common method bias on the research results, this study employed a two-wave, time-lagged survey design.

The first wave (T1) was conducted in early July 2024, during which employees reported their demographic information, empowering leadership, POS, and emotional exhaustion. The second wave (T2) was administered in mid-August 2024, approximately 1 month after T1, measuring retention willingness, proving Go, and avoidance Go. To accurately match responses from the same participant across the two waves while rigorously protecting privacy, an anonymous matching code procedure was implemented. Specifically, at the end of the T1 survey, participants were instructed to create a unique anonymous code by combining the last four digits of their personal mobile phone number with their birth month and day (MMDD format; e.g., code “12,340,501” for a phone ending in “1,234” and a birthday of May 1st). At the beginning of the T2 survey, participants were required to regenerate the identical code.

A total of 428 questionnaires were received across both waves. After screening for invalid responses and matching via the anonymous codes, 387 valid matched questionnaires were retained, yielding a valid response rate of 90.4%. All subsequent analyses were performed on this final sample of 387 participants who completed both surveys, and demographic variables including gender, birth year, education level, work tenure, and job category were analyzed.

Among the valid sample, the gender ratio was relatively balanced (49.1% male, 50.9% female). Most participants were born between 1995 and 2000 (84.5%). Regarding education level, 22% held associate degrees, 59.7% held bachelor’s degrees, and 18.3% held master’s degrees or higher. Industry distribution was relatively even. Positions were concentrated in technology and R&D (36.7%), followed by production and operations (19.1%), marketing and sales (15.0%), and human resources and administration (15.2%). Work tenure distribution was: less than 1 year (28.7%), 1–5 years (60.7%), and over 5 years (10.6%).

### 3.2 Variable measurement

Before completing the questionnaires, participants were informed of the survey’s purpose, and submission was considered informed consent. Participants could withdraw at any time, and personal information was kept confidential.

Established and widely used scales from domestic and international sources were employed. English scales underwent a “translate-back translate” procedure to ensure accuracy. Except for demographic variables, all items used a Likert-5 point scale ranging from 1 (strongly disagree) to 5 (strongly agree).

Empowering leadership: Measured using Ahearne et al.’s (2005) four-dimensional scale (enhancing work meaningfulness, fostering participation in decision-making, expressing confidence in high performance, providing autonomy), with 3 items per dimension (12 items total). Classic questions include: “My leader helps me understand the importance of my work to the overall effectiveness of the company.” This scale is completed by subordinates evaluating their superiors’ behaviors. In this study, the Cronbach’s *α* coefficient was 0.94.

Retention willingness: This study adopted the single-dimensional scale developed by Armstrong-Stassen and Ursel (2009) to measure the retention willingness of Generation Z knowledge workers. A sample item is: “I will continue to develop in my current industry.” In this study, the Cronbach’s *α* coefficient was 0.857.

Perceived organizational support: This study applied Ling et al. (2006) to divide POS into three dimensions: work support, care for interests, and value recognition (16 items total). A sample item is: “The company will agree to my reasonable requests to change my working conditions.” In this study, the Cronbach’s *α* coefficient is 0.95.

Emotional exhaustion: This study used Maslach and Jackson's (1981) single-dimensional scale to measure emotional exhaustion, with a total of eight questions. Sample items include: “Working all day is really a strain for me,” “I feel emotionally drained from my work.” In this study, the Cronbach’s α coefficient was 0.931.

Proving goal orientation: This study used a single-dimensional scale developed by Vandewalle (1997) to measure this, including four items, such as “I really want to prove to others that I perform better than my colleagues.” In this study, the Cronbach’s α coefficient was 0.855. Avoidance goal orientation: This study used a single-dimensional scale developed by Vandewalle (1997) to measure this, including four items. An example item is “I avoid tasks that may make me look incompetent.” In this study, the Cronbach’s α coefficient was 0.854.

Control variables: As questionnaires involved self-reports by employees, and drawing on existing research on empowering leadership (Cheong et al., 2016; Vecchio et al., 2010) and retention intention, this study controlled for the effects of gender, birth year, education level, work tenure, and position category.

## 4 Manuscript formatting

### 4.1 Descriptive statistics and correlation analysis

Means, standard deviations, and correlation coefficients for each variable are presented in Table 1. Table 1 shows that empowering leadership is significantly positively correlated with POS (*r* = 0.280, *p* < 0.01) and emotional exhaustion (*r* = 0.332, *p* < 0.01), and POS is significantly positively correlated with retention willingness (*r* = 0.195, *p* < 0.01). Emotional exhaustion is significantly negatively correlated with retention willingness (*r* = −0.331, *p* < 0.01). The above results provide preliminary support for further hypothesis testing.

**Table 1:** Means, standard deviations, and correlation coefficients of variables (*N* = 387).

Variable	1	2	3	4	5	6	7	8	9	10	11
1 Gender											
2 Year of birth	0.035										
3 Educational level	0.009	−0.141**									
4 Job category	−0.049	−0.066	0.070								
5 Years of experience	0.045	−0.019	−0.019	0.003							
6 Empowering leadership	0.050	0.127*	−0.056	−0.030	0.014						
7 Intention to remain	−0.059	0.062	0.000	−0.020	−0.145**	0.105*					
8 Perceived organizational support	0.020	−0.024	−0.020	0.036	0.030	0.280**	0.195**				
9 Emotional exhaustion	0.000	−0.020	−0.029	−0.069	0.046	0.332**	−0.311**	−0.039			
10 Proving goal orientation	0.013	0.098	−0.060	−0.003	0.053	0.053	0.158**	0.153**	−0.139**		
11 Avoidance goal orientation	−0.050	−0.009	0.022	0.033	−0.034	0.068	−0.189**	−0.109*	0.184**	−0.104*	
Mean	1.509	1.798	1.964	2.506	2.245	3.439	3.315	3.060	3.398	3.270	3.027
Standard deviation	0.501	0.687	0.635	1.460	0.987	1.047	0.951	0.896	1.155	1.134	1.171

*N* = 387, *p < 0.05, **p < 0.01.

### 4.2 Confirmatory factor analysis

This study employed confirmatory factor analysis (CFA) using AMOS 26.0 to examine the discriminant validity of the proposed model. To ensure an appropriate ratio between sample size and parameters, item parceling was applied to two multi-dimensional constructs: Empowering Leadership and POS. Empowering Leadership comprised four dimensions – Meaning, Autonomy, Participation in Decision-Making, and Confidence in High Performance – each with three items averaged into parcels as observed indicators. POS included three dimensions – Work Support (7 items), Value Recognition (4 items), and Care for Interests (5 items) – averaged similarly. This dimension-based parceling reduces model complexity and random error while preserving conceptual coherence.

While item parceling can enhance overall model fit and parsimony by reducing random measurement error and model complexity, it may also obscure item-level variance or mask localized areas of model misfit. To mitigate these potential limitations, a dimension-based, theoretically driven parceling strategy was adopted, rather than arbitrary item combination. Additionally, the internal consistency of each parcel was examined prior to model estimation to ensure conceptual coherence and minimize parceling-induced bias. The results are shown in Table 2. Compared with the other factor, the six-factor model showed the best fit (*χ*^2^ = 343.625, df = 284, *χ*^2^/df = 1.21, CFI = 0.989, GFI = 0.936, NFI = 0.944, RMSEA = 0.023), indicating good fit between the six latent variables.

**Table 2:** Results of confirmatory factor analysis.

Model	χ^2^	df	χ2/df	RMSEA	CFI	GFI	NFI
Six-factor model	343.625	284	1.21	0.023	0.989	0.936	0.944
Five-factor model 1	898.194	289	3.108	0.074	0.896	0.834	0.855
Five-factor model 2	1063.422	289	3.68	0.083	0.868	0.827	0.828
Five-factor model 3	1156.803	289	4.003	0.088	0.852	0.816	0.813
Five-factor model 4	1302.54	289	4.507	0.095	0.827	0.755	0.789
Four-factor model 1	1549.976	293	5.29	0.105	0.786	0.729	0.749
Four-factor model 2	1712.912	293	5.846	0.112	0.758	0.723	0.723
Four-factor model 3	1811.197	293	6.182	0.116	0.741	0.715	0.707
Four-factor model 4	1958.132	293	6.683	0.121	0.716	0.671	0.684

*N* = 387, Six-factor model: each variable corresponds to one factor; Five-factor model 1: POS and retention willingness are combined into one factor; Five-factor model 2: empowering leadership and POS are combined into one factor; Five-factor model 3: POS and emotional exhaustion are combined into one factor; Five-factor model 4: empowering leadership and emotional exhaustion are combined into one factor; Four-factor model 1: POS and retention willingness are combined into one factor, proving goal orientation and avoidance goal orientation are combined into one factor; Four-factor model 2: empowering leadership and POS are combined into one factor, and proving goal orientation and avoidance goal orientation are combined into one factor; Four-factor model 3: POS and emotional exhaustion are combined into one factor, and proving goal orientation and avoidance goal orientation are combined into one factor; Four-factor model 4: empowering leadership and emotional exhaustion are combined into one factor, and proving goal orientation and avoidance goal orientation are combined into one factor.

### 4.3 Common method bias

This paper uses Harman’s single factor test to test for common method bias. Using unrotated principal component analysis, six unrotated factors with eigenvalues greater than 1 were extracted. When unrotated principal components yielded multiple factors and the first factor explained less than 40% of the variance, no serious common method bias was considered to exist (Podsakoff et al., 2003). The results indicate that the variance explained by the first unrotated factor is 23.44%, which is less than the critical value of 40%. Therefore, the common method bias of the variables in this study is not serious.

### 4.4 Hypothesis testing

**Figure 2:**
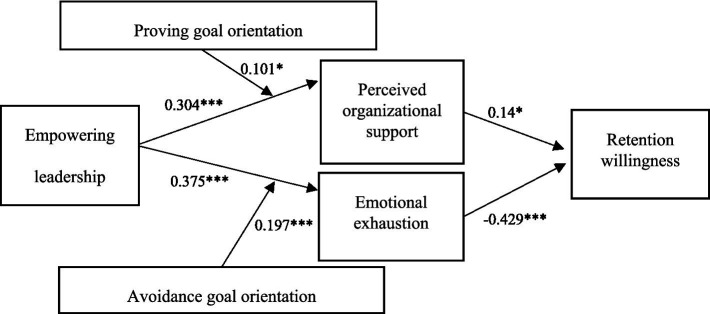
Standardized path coefficients of the model. *p < 0.05, ***p < 0.001.

Path analysis was performed in this study using structural equation modeling (SEM) in Amos 26.0. Path coefficients are shown in Table 3; standardized path coefficients are depicted in Figure 2. The standardized path coefficient from empowering leadership to POS was 0.304 (*p* < 0.001), indicating that empowering leadership has a significant positive effect on POS, i.e., the higher the empowering leadership, the higher the POS. The standardized path coefficient of POS on retention willingness is 0.140 (*p* < 0.05), indicating that POS has a significant positive effect on retention willingness, i.e., the higher the POS, the higher the retention willingness.

**Table 3:** Summary of model coefficients (*N* = 387).

Path	Standardized coefficient	S. E.	C. R.	*P*
Empowering leadership → Perceived organizational support	0.304	0.043	5.628	***
Empowering leadership → Emotional exhaustion	0.375	0.057	6.988	***
Perceived organizational support→ Retention willingness	0.140	0.063	2.463	*
Emotional exhaustion → Retention willingness	−0.429	0.051	−6.925	***
Empowering leadership → Retention willingness	0.232	0.055	3.721	***
Interaction item 1 → (Proving Goal Orientation x Empowering Leadership) → Perceived organizational support	0.101	0.031	2.027	*
Interaction item 2 → (Avoidance of Goal Orientation × Empowering Leadership) → Emotional exhaustion	0.197	0.040	4.109	***

*N* = 387, *p < 0.05, ***p < 0.001.

The standardized path coefficient from empowering leadership to emotional exhaustion was 0.375 (*p* < 0.001), indicating that empowering leadership has a significant positive effect on emotional exhaustion, i.e., the higher the empowering leadership, the higher the emotional exhaustion. The standardized path coefficient from emotional exhaustion to retention willingness is −0.429 (*p* < 0.001), indicating that emotional exhaustion has a significant negative effect on retention willingness, i.e., the higher the emotional exhaustion, the lower the retention willingness.

Taken together, these findings suggest that empowering leadership may simultaneously enhance employees’ retention willingness through increased POS while undermining it through heightened emotional exhaustion. This reflects not merely a statistical outcome but the inherent dual-pathway nature of empowering leadership. The subsequent analysis further unpacks this “double-edged sword” effect by examining the mediating and moderating mechanisms involved.

Additionally, to test the hypothesis of the mediating effect, bootstrapping (2,000 samples, 95% confidence level) was adopted in this study. A significant effect exists if the confidence interval (CI) does not include zero. Results are shown in Table 4. Empowering leadership has a positive and significant indirect effect on retention willingness through POS (indirect effect = 0.037), with a confidence interval of [0.004, 0.082]. The confidence interval does not include 0, indicating that the mediating effect is significant. Therefore, hypothesis 1 is valid. Empowering leadership has a negative and significant indirect effect on retention willingness through emotional exhaustion (indirect effect = −0.141), with a confidence interval of [−0.21, −0.087]. The confidence interval does not include 0, and the mediating effect is significant. Hypothesis 2 is verified.

**Table 4:** Bootstrap mediation effect sizes and confidence intervals (*N* = 387).

Pathway relationship	Direct effect	Indirect effect	95% confidence interval		*p*
			Lower limit	Upper limit	
Empowering Leadership →Perceived organizational support → Retention willingness	0.203	0.037	0.004	0.082	0.026
Empowering Leadership →Emotional exhaustion → Retention willingness	0.203	−0.141	−0.21	−0.087	0.001

**Figure 3:**
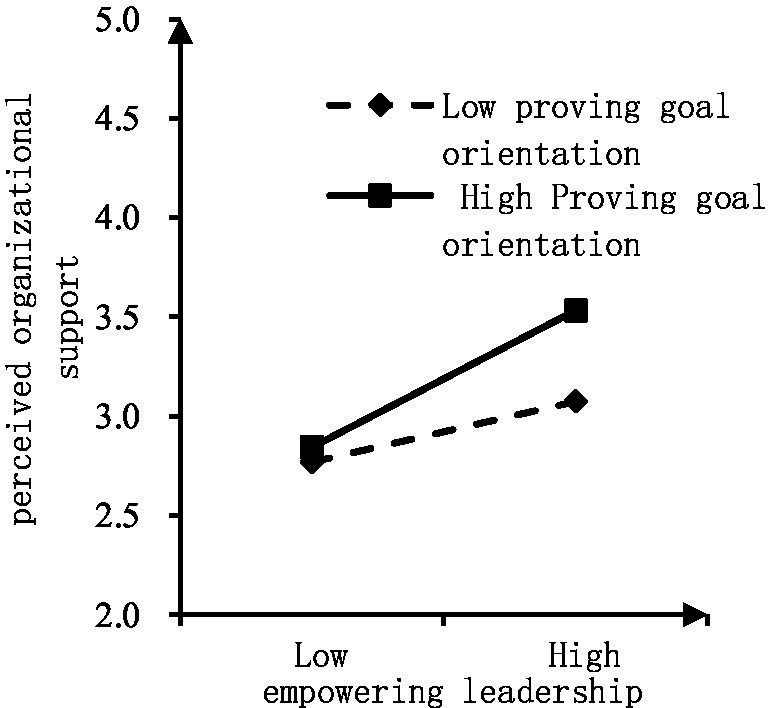
Moderation effect diagram of proving goal orientation on empowering leadership and perceived organizational support.

Regarding the moderating effect of GO on empowering leadership, POS, and emotional exhaustion, Table 3 shows that the standardized path coefficient of interaction term 1 is 0.101, and *p* < 0.05. The path coefficient of the proving GO interaction term is significantly positive, indicating that the moderating effect is valid, i.e., proving GO has a positive moderating effect between empowering leadership and POS. The higher the proving GO, the stronger the empowering leadership and the stronger the employees’ POS. Hypothesis 3 of this study is verified. To visualize this moderating effect, a moderation effect diagram was constructed. As can be seen from Figure 3, the higher the proving GO, the stronger the relationship between empowering leadership and POS.

**Figure 4:**
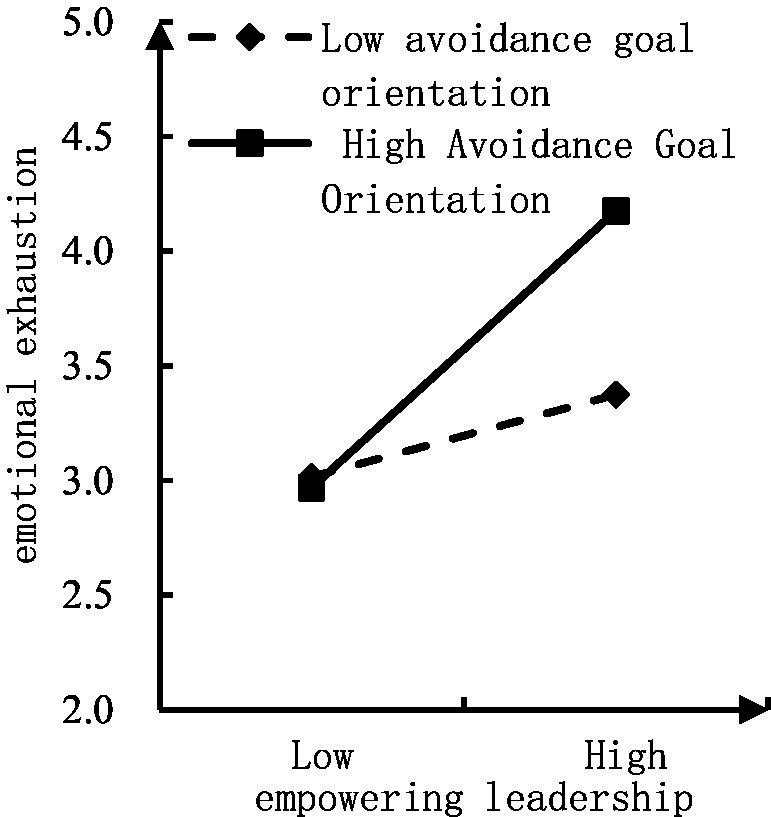
Moderation effect diagram of avoidance goal orientation on empowering leadership and emotional exhaustion.

Similarly, regarding the moderating effect of avoidance GO on the relationship between empowering leadership and emotional exhaustion, Table 3 shows that the path coefficient of interaction term 2 is 0.197, *p* < 0.001, and the interaction term coefficient of avoidance GO is significant, indicating that the moderating effect is established, i.e., avoidance GO has a positive moderating effect on the relationship between empowering leadership and emotional exhaustion. The higher the avoidance GO, the stronger the empowering leadership on employees’ emotional exhaustion. Hypothesis 4 of this study has been verified. To visualize this moderating effect, a moderation effect diagram was constructed. As can be seen from Figure 4, the higher the avoidance GO, the stronger the relationship between empowering leadership and emotional exhaustion.

## 5 Discussion

The findings of this study reveal an intriguing duality in the effects of empowering leadership on employees’ retention willingness. On the one hand, empowering leadership enhances employees’ POS, which in turn strengthens their retention willingness; on the other hand, it simultaneously increases emotional exhaustion, which weakens retention willingness. At first glance, these opposing outcomes may appear contradictory. However, such ambivalence reflects the inherently complex and context-dependent nature of empowerment as a leadership behavior. Drawing upon COR theory and the Job Demands–Resources (JD-R) model, these results can be understood as the simultaneous activation of a resource gain spiral and a resource loss spiral. Empowerment provides employees with autonomy and recognition that foster resource acquisition, yet it also imposes heightened responsibility and psychological demands that can deplete resources.

More importantly, the present study demonstrates that this tension is not universal but contingent upon individual characteristics—specifically, employees’ goal orientation. Individuals with a strong proving GO are more likely to interpret empowerment as a developmental resource, amplifying its positive impact on POS. Conversely, those with a high avoidance GO perceive empowerment as a source of stress, thereby intensifying emotional exhaustion. This person-centered perspective not only reconciles the apparent contradiction but also highlights the crucial role of individual differences in shaping the outcomes of empowering leadership.

### 5.1 Theoretical significance

First, this study provides a unified and theoretically grounded explanation for the “double-edged sword” effect of empowering leadership by integrating the JD-R model with Conservation of Resources (COR) theory, thereby clarifying the contradictory findings reported in the extant literature. Prior inconsistencies regarding the relationship between empowering leadership and employees’ retention willingness largely stem from a predominant focus on a single, typically positive, perspective (Kim and Beehr, 2020). Drawing on COR theory, this study reveals that empowering leadership simultaneously activates both a resource gain spiral and a resource loss spiral.

More critically, this dual-pathway mechanism is explicitly delineated through the theoretical lens of the JD-R model. The enhancement of POS reflects the “motivational process” in the JD-R framework: empowering behaviors are interpreted by employees as valuable job resources that fulfill basic psychological needs, boost energy and engagement, and in turn, increase retention willingness through a resource gain spiral. Conversely, the exacerbation of emotional exhaustion represents the “health-impairment process”: the same empowering behaviors (e.g., increased responsibility and accountability pressure) may also be perceived as job demands. Sustained physical and psychological effort to meet these demands depletes employees’ energy and resources, thereby fostering emotional exhaustion and ultimately reducing retention willingness through a resource loss spiral.

Second, this study addresses the long-standing paradox of empowering leadership by identifying employees’ goal orientation as a pivotal boundary condition. The seemingly contradictory outcomes of empowering leadership can be explained by individual differences in resource appraisal, as posited by COR theory. Empirical evidence demonstrates that goal orientation functions as a critical psychological switch determining which pathway predominates. For employees with a strong proving goal orientation—those motivated to demonstrate competence—empowering leadership is appraised as a valuable resource and developmental opportunity, thereby reinforcing the positive POS pathway. In contrast, employees with a strong avoidance goal orientation—those driven by the desire to evade failure—perceive the same leadership behaviors as demanding and threatening, which amplifies the negative emotional exhaustion pathway (Zhang et al., 2023). This finding effectively integrates trait activation theory into the COR–JD-R framework, offering a precise, person-centered explanation for why identical leadership behaviors elicit divergent employee responses. In doing so, it provides a theoretically grounded resolution to the ambivalence frequently observed in prior empowering leadership research.

Third, this study offers a substantive theoretical and methodological contribution by developing an integrated framework that incorporates dual mediating pathways and a key moderating variable, thereby advancing research on both empowering leadership and COR theory. Whereas prior COR-based studies have typically examined either the resource-gain or resource-loss pathway in isolation, the present model concurrently captures the parallel mediating mechanisms of perceived organizational support (resource gain) and emotional exhaustion (resource loss). This dual-pathway approach provides a more nuanced and ecologically valid understanding of employees’ psychological dynamics when responding to complex work stimuli such as empowerment. Moreover, by identifying goal orientation as a significant moderator, this study systematically integrates a stable individual disposition into leadership-effectiveness frameworks, thereby addressing a critical theoretical gap concerning how personal traits shape responses to empowerment (Cheong et al., 2016; Lee et al., 2017). Collectively, these contributions refine the boundary conditions of empowering leadership’s effectiveness and establish a validated analytical paradigm for exploring other dispositional moderators within the COR and JD-R theoretical frameworks.

### 5.2 Management implications

First, this research provides leaders with a nuanced framework for implementing empowering leadership in a sustainable manner, offering new insights into the scientific empowerment of Generation Z knowledge workers. On the one hand, organizations should be willing to empower, using empowering leadership behaviors to enhance employee autonomy and responsibility, thereby stimulating intrinsic motivation to boost POS and job satisfaction. On the other hand, organizations must be skilled at delegating authority, ensuring that delegation is appropriate and avoiding over- or under-delegation. Organizations need to recognize the potential adverse effects of empowerment on employees and should take appropriate measures to reduce employees’ workload and emotional fatigue to support their professional growth. When employees perceive ongoing opportunities for growth and development within the organization, they are more likely to stay and grow alongside the organization.

Second, it provides new solutions to effectively improve the retention willingness of Generation Z knowledge workers. On the one hand, considering the characteristics of Generation Z knowledge workers themselves, organizations should consistently monitor employees’ workload and work-life balance, providing necessary resources and support to reduce emotional exhaustion. On the other hand, organizations should enhance employees’ POS. Empowering leadership should support and encourage employees, allowing them to feel that the organization values and supports their development. Organizations should establish a positive work climate and create an open, trusting, and cooperative work environment. This positive atmosphere helps strengthen emotional bonds among employees and improves their overall well-being, thereby increasing their retention intention.

Third, it provides a new theoretical basis for achieving “job-person fit” in management practice. To achieve “job fit,” it is necessary to conduct in-depth research on the personal goal characteristics of employees and the job characteristics of the position. In terms of employees’ personal characteristics, this study finds that different GOs (proving GO and avoidance GO) have a moderating effect on the mechanism of empowering leadership and employee retention willingness. Organizations should know their people and assign suitable authority and tasks based on individual traits and capabilities. For employees with high prove performance GO, organizations can set challenging goals and publicly recognize their achievements in team meetings or company announcements to achieve motivational and supportive effects. For employees with high avoid performance GO, leaders need to ensure they receive necessary guidance and support resources for work tasks and can assign them to team projects where peer support and collaboration can share the pressure, reducing their fear and stress regarding personal failure.

### 5.3 Research limitations and outlook

Based on previous studies, this paper constructed a model of the relationship between empowering leadership, POS, emotional exhaustion, proving GO, avoidance GO, and retention willingness among knowledge workers in the knowledge economy. It drew certain conclusions and insights. However, some limitations and shortcomings remain, which are expected to be addressed in future research.

First, the measurement and data collection approach adopted in this study has certain limitations. All six core variables—empowering leadership, POS, emotional exhaustion, proving Go, avoidance GO, and retention willingness—were measured using self-reported data collected from employees. Although appropriate precautions were taken to mitigate common method bias, potential endogeneity cannot be fully ruled out. Future research may employ a matched leader–employee design or utilize multi-source and longitudinal data to strengthen causal inference. In addition, expanding the sample size and extending the investigation across different industries, ownership types, and regions would enhance the external validity and generalizability of the results.

Second, regarding the measurement tools and scales, this study mainly relied on instruments that were originally developed and validated in Western contexts. While these scales are mature and widely used, cultural differences may influence respondents’ understanding of certain items, thereby affecting construct validity. Future research should not only select scales with proven psychometric properties but also pay attention to cultural adaptation and localization. Specifically, researchers are encouraged to develop or refine measurement tools that reflect indigenous Chinese values—such as relational harmony, collective orientation, and hierarchical respect—so as to improve measurement accuracy, contextual relevance, and cultural sensitivity. Moreover, given that social and organizational environments evolve rapidly, it is advisable to periodically reassess the validity of these instruments to ensure they remain theoretically and culturally up to date.

Third, the contextual and theoretical boundary of this study presents both a strength and a limitation. The data were collected from Chinese SMEs, a setting characterized by relatively high power distance, relational interdependence, and performance pressures faced by young knowledge workers. These unique institutional and cultural features—particularly the differential mode of association and relationship-oriented management logic—may have significantly shaped how Gen Z employees perceive and react to empowering leadership. Within such a context, empowerment is often filtered through the lens of human sentiment and hierarchical norms, giving rise to distinct patterns of relational empowerment (based on interpersonal trust and emotional exchange) and task empowerment (based on autonomy and performance expectations). This nuanced dynamic helps explain why empowering leadership may yield ambivalent outcomes: it can simultaneously foster psychological safety and induce social pressure, depending on how employees interpret relational obligations. While these features enhance the contextual richness of the findings, they also constrain their cross-cultural generalizability.

Future studies could therefore conduct comparative or cross-cultural investigations to examine whether the dual mechanisms of empowering leadership—resource gain via POS and resource loss via emotional exhaustion—operate similarly under different cultural or institutional logics. Such efforts would help verify the robustness and universality of the proposed model and provide a valuable foundation for expanding empowering leadership research beyond the Chinese context. Additionally, future work may further explore multi-level mechanisms (e.g., team or organizational levels) and alternative mediators or moderators (e.g., relational versus task-based empowerment climates) to deepen the theoretical understanding of empowering leadership’s dynamic effects on employee attitudes and behaviors.

## 6 Conclusion

Based on the COR theory, this study constructed a research framework and theoretical model to explore the intrinsic mechanisms and boundary conditions of empowering leadership on the retention intention of Generation Z knowledge workers. Data analysis led to the following conclusions: (1) Empowering leadership significantly and positively affects POS and significantly and positively affects emotional exhaustion. POS significantly and positively affects retention willingness, while emotional exhaustion significantly and negatively affects retention willingness. (2) POS and emotional exhaustion mediated the relationship between empowering leadership and retention intention, respectively. (3) Employee GO moderates the effect of empowering leadership: employees with high proving GO increase their POS under empowerment, while employees with high avoidance GO exacerbate emotional exhaustion. This shows that the effect of empowerment varies depending on employee GO. Therefore, in practical management, organizations should tailor empowerment scientifically based on industry, position, and employee traits to achieve role fit, thereby enhancing retention intention and reducing human capital loss.

## Data Availability

The original contributions presented in the study are included in the article/supplementary material, further inquiries can be directed to the corresponding author.
